# Research on the influence of sports participation on school bullying among college students—Chain mediating analysis of emotional intelligence and self-esteem

**DOI:** 10.3389/fpsyg.2022.874458

**Published:** 2022-09-28

**Authors:** Ouyang Yiyi, Peng Jie, Luo Jiong, Teng Jinsheng, Wang Kun, Li Jing

**Affiliations:** ^1^College of Physical Education, Chongqing University of Posts and Telecommunications, Chongqing, China; ^2^College of Physical Education, Liupanshui Normal University, Liupanshui, China; ^3^College of Physical Education, Southwest University, Chongqing, China; ^4^College of Physical Education, Sichuan University, Chengdu, China

**Keywords:** sports participation, school bullying, emotional intelligence, self-esteem, mediating role

## Abstract

**Purposes:**

This paper aims to discuss the relationship between college students’ sports participation, school bullying, emotional intelligence and self-esteem. At the same time, it explores the intrinsic mechanisms of school bullying, in order to provide a reference for reducing bullying phenomenon among college students, and pave the way for college students to lead happy, healthy and confident lives.

**Methods:**

A total of 1,317 students (725 male students, 592 female students, 21.31 ± 3.28 years old) from four universities in Southwest China were selected as subjects for this survey. They were selected by stratified random sampling, and the data needed was obtained using a structured questionnaire. The data was subsequently processed with statistical software SPSS19.0 and AMOS21.0.

**Results:**

(1) Sports participation has a significant and positive correlation with emotional intelligence and self-esteem, likewise, it has a significant negative correlation with school bullying. Emotional intelligence has a marked positive correlation with self-esteem, at the same time it has a significant negative correlation with school bullying. In addition, self-esteem is strongly negative correlated with school bullying. (2) Sports participation has a direct effect on school bullying (ES = −0.271). Emotional intelligence (ES = −0.144) and self-esteem (ES = −0.065) also play a significant mediating role between sports participation and school bullying, and the chain mediating force of emotional intelligence—self-esteem also reaches a significant level (ES = −0.016).

**Conclusion:**

Sports participation affects school bullying among college students not only directly but also indirectly, such as through emotional intelligence, the mediating role of self-esteem, and the chain mediating role of emotional intelligence to self-esteem. Apart from sports participation, emotional intelligence is another key factor that affects college students’ school bullying. Therefore, while attaching great importance to college students’ sport participation, schools should also provide courses aimed at developing students’ emotional intelligence.

## Introduction

School bullying refers to deliberate or malicious bullying incidents that occur on campus, which are imposed repetitively on students by other individual students or groups for specific reasons. This deviant behavior of the individual or group is intended to cause physical or mental damage to the bullied student (Zhu, 2018; Zhao et al., 2020), such as bullying, insulting or injuring either physically, verbally or virtually, etc. At the same time, the object of school bullying is often fixed, and the practice of school bullying has the essential characteristics of abusing the weak, intentional injury, repetition, and power imbalance. There are typically varying degrees of impact and harm on the body and mind of bullies, victims and bystanders in the long run. These effects include distorting their views on education and society, changing their normal behaviors, or even lead to long-term anxiety, depression, and life loss or abandonment among the bullied. Bullying also has a retaliative nature (MOE, 2017; Hao et al., 2019; Zhao et al., 2020). Thus, school bullying is a serious social problem, which has adverse physical and mental effects on both the bullies and the bullied.

Without effective counseling, the bullies in school bullying incidents may become more involved in social violence in the future, and bullying others also increases an individual’s risk of alcoholism, drug use, delinquency, and the probability of crime (one or more crimes) in their adulthood behavior (Bender and Losel, 2011; Farrington and Ttofi, 2011). The bullied person may develop a strong sense of anxiety, depression, loneliness, helplessness, or even an aversion to learning, personality disorders or a tendency to suicide in the long-term, in addition to other extreme consequences (Benedict et al., 2015; Chan and Wong, 2015; Liu, 2018). There are many factors that lead to school bullying, mainly in regard to individuals, families, schools and society at large (Pistella et al., 2020; Ringdal et al., 2020; Arslan et al., 2021; Xu et al., 2022). However, personal factors remain the key target of investigation for current research on school bullying.

In recent years, some scholars have found (Moore et al., 2019; Holbrook et al., 2020; Nikolaou and Crispin, 2022) that sports participation can reduce the occurrence of school bullying, and have also confirmed that sports participation is an effective intervention strategy, in that it adopts physical exercise to promote positive peer interaction and emotions, thereby serving as an effective strategy to reduce school bullying. The Embodied Cognition Theory (Ye et al., 2019) holds that the mind is a result of our body, therefore changing one’s body posture changes their psychological perception. Exercise is a direct way to influence the body, triggering more open body postures, which then changes an individual’s feelings over a brief period of time (Wang, 2021). Studies have found that long-term sports participation can improve an individual’s emotion regulation ability, strategy and belief which can reduce rebellious teenage behavior and improve their self-control, which is an important factor to improve peer relationships (Zhu et al., 2017; Xie C. Y. et al., 2019).

Self-control and peer relationships are important predictive factors of school bullying (Wang, 2021). Therefore, some scholars have proposed a question—can sports participation reduce the occurrence of school bullying? Additionally, according to relevant research reports, an individual’s emotional recognition ability, information processing ability and self-control ability are important variables for predicting school bullying (Huang et al., 2019; Kowalski et al., 2019; Xie J. S. et al., 2019; Wang et al., 2021). These variables are closely related to the brain’s executive functions, which include emotion management, self-monitoring, working memory, inhibitory control and attentional flexibility. At the same time, executive function is not only influenced by individual’s social experience, but also affects individual’s social result. Bullying is a kind of social interaction event, where differences exist in the executive function between the bullies and the victims. That is, the bullies tend to use hostile stimuli in the process of decision-making and may interpret ambiguous situations in a hostile way, while the victims show poor levels of behavioral inhibition and cognitive flexibility (Medeiros et al., 2016; Jenkins et al., 2018).

Therefore, an individual’s executive function affects the developmental trajectory of a school bullying incident. In recent years, however, an increasing number of researches have explored school bullying through individual sports participation, and it has become a new research focus. Participating in exercise can effectively regulate neurotransmitter and hormone levels, improve brain function and its functional network, and have a positive effect on individual executive function – there is a positive causal relationship between the two (Wen, 2015; Chang et al., 2017; Yin et al., 2018). Along with the enhancement of individual executive function, the behavior control ability of the bully and the cognitive flexibility and emotional regulation ability of the bullied person will all be improved. Under the influence of this combined force, the occurrence of school bullying incidences can be effectively reduced (Pietromonaco and Collins, 2017; Pietromonaco and Beck, 2019; Wang, 2021). Therefore, sports participation can effectively reduce the occurrence of school bullying.

Several studies have found that emotional intelligence and self-esteem play a preventative role against school bullying (Zhao et al., 2018; Canas et al., 2020b; Wang, 2021). Emotional intelligence refers to the ability of an individual to perceive, evaluate and express emotions accurately, the ability to generate emotions that promote thinking, the ability to understand emotions and emotional knowledge and the ability to regulate emotions and promote one’s emotional and intellectual development (Li and Wang, 2021). From the perspective of the social control theory, the more importance an individual attaches to the expectations and opinions of others, the less likely he is to have deviant behavior.

However, if an individual does not care about the opinions of others around him and does not want to conform to others’ expectations, he is more prone to be bullied (Zhang Y. Y. et al., 2021). By improving emotional intelligence, the individual’s ability to regulate and understand emotions and interpersonal communication skills will be improved, thereby reducing the occurrence of bullying (Peachey et al., 2017; Canas et al., 2020b). Self-esteem is an emotional evaluation of an individual’s positive or negative self, and it is an important factor in predicting the implementation of healthy behaviors (Ouyang et al., 2020). Many scholars believe that self-esteem has a negative predictive effect on school bullying.

People with low self-esteem often have relatively low self-evaluation and sense of self-worth, and may not be able to resolutely defend themselves against bullying or protect their own legitimate rights and even personal dignity. They choose to make compromises blindly instead, thus giving birth to constant school bullying incidents. People with higher self-esteem have more mature and stable self-evaluation and sense of self-worth, and are better than those with lower self-esteem in their mastery of interpersonal skills and management of negative emotions. They can promote the improvement of peer relationships, thereby avoiding the occurrence of school bullying (Malecki et al., 2015; Yan et al., 2019; Li and Hu, 2020). In addition, there is a positive correlation between emotional intelligence and self-esteem. People with high emotional intelligence often have high self-esteem, and vice versa (Ding and Ma, 2013; Garaigordobil, 2020; Li, 2020). All in all, emotional intelligence and self-esteem have a positive effect on school bullying prevention, and there is a positive relationship between the two.

Several studies have found that emotional intelligence and self-esteem are influenced by an individual’s sports participation. Because exercise can promote the increases of an individual’s enkephalin, serotonin, dopamine and norepinephrine, it can promote the improvement of one’s emotion regulation ability and belief. At the same time, positive emotion regulation strategies also have a positive impact on individuals’ emotional intelligence (Gáspár et al., 2017; Mendez-Gimenez et al., 2022); meanwhile, participating in sports can promote individuals to form stable self-esteem, thereby improving their self-esteem level (Guddal et al., 2019; Qurban et al., 2019; Bang et al., 2020); in addition, sports is a type of social interaction, and regular participation in sports can not only improve an individual’s self-confidence in social interactions, but also help improve his interpersonal relationships. In interpersonal interaction, one can learn to perceive and control both his own emotions as well as other people’s emotions (Zhu et al., 2016, 2017; Moore et al., 2019).

In addition, some studies found that (Ren et al., 2014; Wang, 2021; Zhang Y. Y. et al., 2021) sports participation can effectively improve the executive function of the brain, promoting the emotional intelligence, self-esteem and personality development of individuals, thus improving peer relationships and reducing the occurrence of school bullying. This seems to prove that sports participation can influence school bullying by improving students’ emotional intelligence or self-esteem, and it may also imply that emotional intelligence and self-esteem play a mediating role between sports participation and school bullying.

On this basis, we conclude that sports participation can affect school bullying through emotional intelligence or self-esteem, and there is also a positive correlation between emotional intelligence and self-esteem. This seems to imply that an increase in college students’ sports participation will improve their emotional intelligence and make it easier for them to develop stable self-esteem. Is sports participation the key factor then in tackling school bullying? In previous studies, the research on school bullying mainly focuses on primary and secondary schools, and few scholars have discussed school bullying at the university level. So, is there also school bullying in the relationship between college students? Likewise, very few scholars have discussed the relationship between “sports participation, emotional intelligence, self-esteem and school bullying,” and this topic especially lacks in-depth research on whether the chain mediating mechanism of “emotional intelligence + self-esteem” exists.

With this in mind, this study takes college students as the subject, and constructs a chain mediating model of sports participation, emotional intelligence, self-esteem and school bullying. We take sports participation, emotional intelligence and self-esteem as a personality system, and integrate the three factors to examine their influence on school bullying to reveal the relevant mechanism that deeply influences college students’ school bullying. Finally, we provide corresponding coping strategies for schools, parents and students, and provide practical reference for enhancing students’ physical and mental health. So far, the following hypotheses have been put forward: ➀ Emotional intelligence acts as a mediator between sports participation and school bullying; ➁ Self-esteem acts as a mediator between sports participation and school bullying; ➂ Emotional intelligence and self-esteem play a chain mediating role between sports participation and school bullying.

## Materials and methods

### Participants

Students enrolled in four universities in Southwest China were selected as the survey subjects (two of the universities are comprehensive universities directly under the Ministry of Education of China, and two are comprehensive universities directly under the local government). The research subjects were first categorized according to their disciplines and grades, and then 400 students were selected from each university by random sampling, and 100 paper questionnaires were distributed to each grade. After the survey was approved by the ethics committee, the selected students were surveyed anonymously with the help of public physical education teachers and college counselors in these universities; those who filled out the questionnaires all volunteered for free. A total of 1,600 questionnaires were distributed, and 1,317 valid questionnaires were recovered, with an effective recovery rate of 82.31%. Among them, a total of 232 questionnaires were excluded, including 133 questionnaires due to missing completed questions, 61 questionnaires because they were not filled in carefully, and 89 questionnaires because they were not returned in time.

The basic information of the sample is as follows (Table 1): gender (725 males and 592 females), place of upbringing (766 were born in urban areas and 551 in rural areas), school type (736 in comprehensive universities directly under the Ministry of Education and 581 in local comprehensive universities), average age of 21.31 ± 3.28 years, height of 174.30 ± 13.46 cm, body weight of 68.38 ± 9.24 kg and BMI of 22.37 ± 3.56. Among them, there were 354 freshmen, 308 sophomore, 336 junior students and 319 senior students. The individuals’ BMI value was converted with the body mass index (BMI) formula: BMI = weight (Kg)\height (m)^2^. According to the fitness BMI standard of college students of the Ministry of Education (Chen et al., 2019), subjects were divided into three groups according to BMI, with the sample size of the underweight group (BMI < 20 kg/m^2^) being 302, that of the normal weight group (BMI = 20–25 kg/m^2^) being 778, and that of the overweight group (BMI > 25 kg/m^2^) being 237.

**TABLE 1:** Basic information of the sample (*N* = 1,317).

Variable	Mean ± SD	Variables	Category	*n*	%
Age (years)	21.31 ± 3.28	Gender	Male	725	55.05%
Height (cm)	174.30 ± 13.46 cm		Female	592	44.95%
Weight (kg)	68.38 ± 9.24 kg	Place of upbringing	City	766	58.16%
BMI (kg/m^2^)	22.37 ± 3.56		Countryside	551	41.84%
		School type	Comprehensive university directly under the ministry of education	736	55.88%
			Local comprehensive university	581	44.12%
		Grade	Freshman	354	26.88%
			Sophomore	308	23.39%
			Junior	336	25.51%
			Senior	319	24.22%
		BMI	Underweight group	302	22.93%
			Normal weight group	778	59.07%
			Overweight group	237	18.00%

BMI, body mass index. BMI = body weight (units in kilograms)/height (units in square meters). Underweight BMI < 20 kg/m^2^, normal BMI: 20–25 kg/m^2^, overweight BMI > 25 kg/m^2^ (BMI grouping according to the Ministry of Education of China criteria for college students).

### Procedures

This study is a descriptive study, using a structured questionnaire as the survey tool, with the required data being obtained through a questionnaire. A two-phase questionnaire was adopted in order to secure a meticulous and effective research. Procedures: First, prepare an initial questionnaire based on the research objective and reference to a large amount of research literature. Second, conduct a small-sample survey of 200 people to test normal distribution verification, reliability analysis and exploratory factor analysis; carry out structural model verification. Finally, settle on the final draft of the questionnaire.

### Instruments

(1) Personal background: The content includes the basic information of subjects’ gender, place of upbringing, school type, grade, height, weight and so on. The body mass index (BMI) of the students is calculated according to their height and weight.
(2) Sports Participation Scale: According to Fox (1999), the scale was developed into a three-question test mode, for example, the intensity of your physical exercise is (); you usually do the above physical exercise () minutes per time; you usually do the above physical exercise () times a month, with sports participation degree = sports frequency × (average sports intensity + sports duration). The score range is 2–72. The higher the score, the higher the students’ sports participation. The exercise frequency is determined by the number of times an individual exercises per week; the numbers 1–6 represent 0, 1, 2, 3, 4, and more than 5 times/weeks, respectively. Exercise duration is determined by the average time spent in each exercise; the numbers 1–6 represent 0–10, 11–20, 21–30, 31–40, 41–50, and ≥ 51 min/time, respectively. The average exercise intensity is determined by the fatigue degree of the subjects after each exercise. Numbers 1–6 represent “very relaxed,” “relatively relaxed,” “relaxed,” “a little tired,” “relatively tired” and “very tired,” respectively. The pretest of the questionnaire shows that the retest reliability is high, and the correlation coefficient *r* = 0.89.
(3) The School Bullying Scale mainly refers to that of Xie et al., 2016, 2018. The Delaware Bullying Victimization Scale-Student (DBVS-S for short) was revised to the 2016 Chinese version, with 17 entries in total. It includes verbal bullying (4 items), physical bullying (4 items), social/relationship bullying (4 items) and online bullying (4 items), with one extra item not included in the dimension score, that is, Article 13 “I was bullied in this school,” which aims to test whether the individuals think that they have been bullied in the above-mentioned bullying situations. At the same time, such a scale also provides a conspicuous way for schools to identify school bullying incidents. The scale is scored on a 6-point Likert scale, where 1 means never, 2 means occasionally, 3 means once or twice a month, 4 means once a week, 5 means more than once a week, and 6 means the incident happens on an everyday basis. The higher the score, the more severe the bullying is in the past year.
  The Cronbach’s α value of the school bullying scale in all dimensions is greater than 0.70 (0.82–0.93), which fully affirms that the scale has good reliability. The verification result of the measurement model of this scale is that X^2^/DF = 1.844, and the values of CFI (comparative fit index), GFI (goodness-of-fit index), AGFI (adjusted goodness-of-fit index), TLI (Tucker–Lewis index), IFI (incremental fit index), and RMSEA (root mean square error of approximation) are 0.995, 0.991, 0.984, 0.992, 0.995, and 0.026, respectively, which all meet the acceptable standards, indicating that the model fits well with the data obtained from the survey and that the scale has good structural validity. Therefore, the school bullying scale has both good reliability and validity.
(4) Emotional Intelligence Scale: The paper adopts the Chinese version of EIS (Emotional Intelligence Scale) of Schutte et al. translated by Wang (Li and Wang, 2021), which is suitable for ordinary adults and teenagers. This scale has 33 items in total, including four dimensions: emotional perception (12 items), self-emotional regulation (8 items), regulation of other people’s emotions (6 items) and emotion utilization (7 items). The scale is scored on a 5-point Likert scale, where “1” means “strongly disagree,” “2” means “disagree,” “3” means “neither agree nor disagree,” “4” means “agree,”, and “5” means “strongly agree.” Of those, Questions 5, 28, and 33 are reverse scored, indicating that the higher the score, the more positive the individual’s emotions, the better their ability is to restrain impulses, the more clearly they express their feelings, and the stronger their ability of self-monitoring and empathy.
  The Cronbach’s α value of the emotional intelligence scale is greater than 0.70 (0.88–0.96) in all dimensions, which fully affirms that the scale has good reliability. The verification results of the measurement model of this scale show that X^2^/DF = 1.405, and the values of CFI, GFI, AGFI, TLI, IFI, and RMSEA are 0.998, 0.994, 0.988, 0.996, 0.998, and 0.018, respectively, which all meet the acceptable standards. This shows that the model fits well with the data obtained from the survey, indicating that the scale has good structural validity. Therefore, the emotional intelligence scale has good reliability and validity.
(5) Self-esteem Scale: This scale is the translated and revised version of the self-esteem scale compiled by Rosenberg (Tian, 2006) and the revised scale is divided into 10 questions. It adopts 4-point scoring, which ranges from completely unqualified (1 point) to fully qualified (4 points). Questions 2, 5, 6, 8, and 9 are reverse questions, and reverse scoring is adopted for these questions. The higher the score is, the more positive the self-esteem will be. The overall Cronbach’s α value of the self-esteem scale is 0.90, which fully affirms that the scale has good reliability. The verification results of the measurement model of this scale show that X^2^/DF = 1.665, and the values of CFI, GFI, AGFI, TLI, IFI, and RMSEA are 0.996, 0.992, 0.985, 0.994, 0.996, and 0.023, respectively, which all meet the acceptable standards. It shows that the model fits well with the data obtained from the survey, indicating that the scale has good structural validity. Therefore, the self-esteem scale has good reliability and validity.

### Data analyses

The data obtained in this study was analyzed using software SPSS19.0 and AM0S21.0. The normal distribution of all variables was examined using Kolmogorov-Smirnov test, and all continuous variables follow the normal distribution. The statistical methods included descriptive statistics, Kolmogorov–Smirnov test, reliability analysis, exploratory factor analysis, Harman single factor test, independent sample *t*-test, one-way ANOVA, correlation analysis, structural equation model, and Bootstrap analysis. The significance level of all variables was set as α = 0.05.

Statistics obtained from questionnaires may lead to common method biases. This research used Harman single factor test to test possible common method biases (Tang and Wen, 2020). The results show that the characteristic roots of a total of 14 factors are greater than 1, among which the largest factor explained variance is 13.45%, far from the critical standard of 40%. It can be seen that the research is less likely to be affected by common method biases, which is within the acceptable range.

## Results

### Comparison of differences in sports participation, school bullying, emotional intelligence and self-esteem by personal background in college students

Table 2 shows the following:

**TABLE 2:** Comparison of differences in sports participation, school bullying, emotional intelligence and self-esteem by personal background in college students (*N* = 1,317).

Variables	Sports participation	School bullying	Emotional intelligence	Self-esteem
**Gender**				
Male	28.83 (11.17)	51.92 (8.77)	117.03 (12.59)	31.27 (8.06)
Female	24.83 (7.39)	53.40 (9.39)	114.96 (10.68)	31.00 (6.82)
*t*–value	5.09***	−0.36	0.84	0.13
**Place of upbringing**				
City	27.31 (10.21)	51.92 (5.70)	119.96 (14.26)	31.12 (7.06)
Countryside	26.50 (8.70)	54.40 (8.85)	115.56 (12.90)	30.89 (6.93)
*t*–value	0.27	−3.11**	4.19***	2.02*
**School type**				
Comprehensive university directly under the ministry of education	26.73 (9.36)	52.02 (7.97)	114.56 (12.22)	31.51 (7.57)
Local comprehensive university	26.13 (9.79)	53.21 (6.64)	116.66 (11.14)	30.94 (6.88)
*t*–value	0.18	−0.21	−0.77	1.53
**Grade**				
Freshman^1^	25.66 (10.31)	53.54 (7.72)	113.56 (15.02)	31.15 (6.98)
Sophomore^2^	25.94 (7.08)	52.24 (6.69)	118.39 (11.14)	30.95 (7.04)
Junior^3^	27.82 (8.99)	52.62 (8.07)	117.51 (13.51)	30.88 (6.89)
Senior^4^	26.64 (9.71)	51.21 (9.44)	119.66 (10.82)	30.38 (6.93)
*F*-value	1.39	1.17	2.20	2.52
**LSD**				
**BMI**				
Light weight^1^	25.91 (8.91)	53.00 (8.12)	117.36 (11.70)	31.04 (6.95)
Normal weight^2^	27.85 (9.69)	50.24 (10.14)	118.13 (11.19)	31.12 (7.05)
Overweight^3^	26.49 (9.99)	53.92 (5.97)	114.66 (14.14)	30.77 (6.72)
*F*-value	4.53**	7.47***	2.49	2.55
LSD	3 > 2;2 > 1	3 < 2;2 < 1		

*p < 0.05; **p < 0.01; ***p < 0.001. t-value: the value of the independent-sample t-test; F-value: the value of the one-way ANVOA test; LSD, Least square difference. The numbers act relative to a reference object for LSD comparison and compare 1, 2, and 3 with LSD.

1. Significant gender differences were found in sports participation (*T* = 5.09, *P* < 0.001), while school bullying (T = −0.36, *P* > 0.05), emotional intelligence (T = 0.84, *P* > 0.05) and self-esteem (T = 0.13, *P* > 0.05) did not correlate with gender. The results show that male students got higher scores than female students in sports participation.
2. School bullying (*T* = −3.11, *P* < 0.01), emotional intelligence (*T* = 4.19, *P* < 0.001) and self-esteem (*T* = 2.02, *P* < 0.05) had significant differences depending on place of upbringing, while sports participation (*T* = 0.27, *P* > 0.05) had no correlation with place of upbringing. The results show that urban college students got higher scores in emotional intelligence and self-esteem than rural college students, but rural college students got higher scores in school bullying than urban college students.
3. The scores in sports participation (*T* = 0.18, *P* > 0.05), school bullying (*T* = −0.21, *P* > 0.05), emotional intelligence (*T* = −0.77, *P* > 0.05) and self-esteem (*T* = 1.53, *P* > 0.05) did not correlate with the type of school.
4. The scores in sports participation (*F* = 1.39, *P* > 0.05), school bullying (*F* = 1.17, *P* > 0.05), emotional intelligence (*F* = 2.20, *P* > 0.05) and self-esteem (*F* = 2.52, *P* > 0.05) did not correlate with grade.
5. Sports participation (*F* = 4.53, *P* < 0.01) and school bullying (*F* = 7.47, *P* < 0.001) were significantly influenced by BMI, but emotional intelligence (*F* = 2.49, *P* > 0.05) and self-esteem (*F* = 2.55, *P* > 0.05) did not seem to correlate with BMI. The results show that normal weight subjects got significantly higher scores in sports participation than both underweight and overweight subjects, but normal weight subjects got significantly lower scores in regards to school bullying than both underweight and overweight subjects.

### Correlation analysis of college students’ sports participation, school bullying, emotional intelligence and self-esteem

Pearson correlation is used to analyze the correlation coefficients among sports participation, school bullying, emotional intelligence and self-esteem (see Table 3). Sports participation is positively correlated with emotional intelligence and self-esteem, but negatively correlated with school bullying. Emotional intelligence is positively correlated with self-esteem, but negatively correlated with school bullying; likewise, self-esteem is negatively correlated with school bullying. In addition, according to the correlation analysis between demographic variables and research variables, it is found that there is no significant correlation between gender, place of upbringing, school type, grade, BMI and other research variables, and they are not controlled in the process of research hypothesis test. The above correlation analysis results provide a basis for testing the subsequent hypotheses.

**TABLE 3:** Correlation analysis table of college students’ sports participation, school bullying, emotional intelligence and self-esteem (*N* = 1,317).

	M ± SD	1	2	3	4	5	6	7	8	9	10	11	12	13	14	15
1. Gender	−	1.00														
2. Place of growth	−	0.04	1.00													
3. School type	−	−0.03	0.34**	1.00												
4. Grade	−	−0.02	−0.01	0.01	1.00											
5. BMI	22.37 ± 3.56	−0.05	−0.03	0.04	0.01	1.00										
6. Sports participation	26.76 ± 9.46	0.05	0.02	0.04	0.02	0.01	1.00									
7. Verbal bullying	15.39 ± 6.38	−0.01	−0.03	0.01	−0.03	0.03	−0.41**	1.00								
8. Physical bullying	9.52 ± 3.15	−0.02	−0.01	0.02	−0.01	0.02	−0.34**	0.56**	1.00							
9. Relationship bullying	14.72 ± 5.78	−0.01	−0.02	0.03	−0.03	0.03	−0.38**	0.58**	0.53**	1.00						
10. Cyberbullying	13.27 ± 4.43	−0.01	−0.01	0.01	−0.04	0.04	−0.32**	0.57**	0.50**	0.51**	1.00					
11. Emotional perception	39.12 ± 4.53	0.01	0.02	0.01	0.04	−0.03	0.26**	−0.33**	−0.29**	−0.30**	−0.31**	1.00				
12. Emotional self-regulation	27.95 ± 3.84	0.04	0.01	0.02	0.03	−0.02	0.30**	−0.34**	−0.28**	−0.31**	−0.29**	0.58**	1.00			
13. Others’ emotion management	23.00 ± 3.80	0.05	0.03	0.02	0.03	−0.02	0.31**	−0.38**	−0.34**	−0.29**	−0.33**	0.63**	0.61**	1.00		
14. Emotional utilization	25.61 ± 4.38	0.03	0.01	0.01	0.05	−0.03	0.25**	−0.32**	−0.30**	−0.25**	−0.29**	0.55**	0.52**	0.57**	1.00	
15. Self-esteem	31.02 ± 7.97	0.05	0.04	0.01	0.04	−0.05	0.44**	−0.37**	−0.029**	−0.34**	−0.31**	0.26**	0.31**	0.30**	0.32**	1.00

*p < 0.05; **p < 0.01.

### Model validation analysis of college students’ sports participation, school bullying, emotional intelligence and self-esteem

**FIGURE 1:**
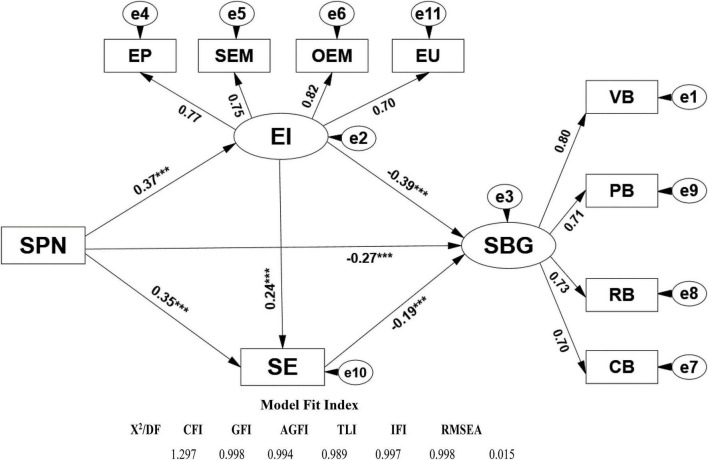
Path analysis diagram and model fitting test of sports participation, school bullying, emotional intelligence and self-esteem. SPN, sports participation; SBG, school bullying; VB, verbal bullying; PB, physical bullying; RB, relationship bullying; CB, cyberbullying; EI, emotional intelligence; EP, emotional perception; SEM, self-emotional management; OEM, others’ emotion management; EU, emotional utilization; SE, self-esteem. *p < 0.05; **p < 0.01; ***p < 0.001.

In order to investigate the relationship of sports participation, school bullying, emotional intelligence, and self-esteem, and to test the mediating role of emotional intelligence and self-esteem, this study used AMOS to make structural equation model analysis of the relationship of sports participation, emotional intelligence, self-esteem and school bullying. The model was corrected to obtain model (Figure 1). The model fitting index is: *X*^2^/DF = 1.297 < 2.000, CFI = 0.998, GFI = 0.994, AGFI = 0.989, TLI = 0.997, IFI = 0.998 > 0.900, and RMSEA = 0.015 < 0.080, indicating that the model could be established.

From the standardized path coefficient β value and significant level in the mixed model structure of Figure 1, it is not difficult to find that sports participation has a significant positive predictive effect on emotional intelligence and self-esteem (β = 0.370^***^, *P* < 0.001; β = 0.352^***^, *P* < 0.001), but there is a significant reverse prediction on school bullying (β = −0.271^***^, *P* < 0.001). At the same time, emotional intelligence and self-esteem have a significant positive predictive effect (β = 0.239^***^, *P* < 0.001), but they have a significant negative predictive effect on school bullying (β = −0.388^***^, *P* < 0.001); self-esteem also has a significant positive predictive effect on sports participation (β = −0.185^***^, *P* < 0.01). In order to verify the mediating role of emotional intelligence and self-esteem in sports participation and school bullying, the non-parametric percentile Bootstrap program was used to test the significance of the mediating role.

The original data was sampled 5,000 times repeatedly, and then the 95% confidence interval (CI) was calculated. If the normalized path coefficient 95% CI does not contain zero, it means that the mediating role is significant. From Table 4, it can be seen that the chain mediating role of sports participation from emotional intelligence and self-esteem to school bullying is 95% CI [−0.021, −0.012]; the mediating role of sports participation from emotional intelligence to school bullying is 95% CI [−0.201, −0.087]; and the mediating role of sports participation from self-esteem to school bullying is 95%CI [−0.056, −0.092]. None of the above three intervals include 0, indicating that each mediating role is significant. Further analysis of the effects of various variables on school bullying showed that the direct effect of sports participation in school bullying is −0.271, and the total mediating effect value (−0.225) is the sum of the mediating effects of the three mediating paths. The total indirect effect, and the sum of the direct effect and the total mediating effect are the total effect, that is, −0.496; the mediating effect value divided by the total effect is the effect quantity, and the effect quantities of the three mediating paths in this paper are 3.23, 29.03, and 13.10%, respectively. Therefore, it can be seen that hypotheses 1–3 are all valid.

**TABLE 4:** Path and effect analysis table of sports participation on school bullying.

Effect	Path relationship	Effect size	Bootstrap SE	Bootstrap 95% CI	Relative mediating effect (%)
Direct effect	Sports participation → school bullying	−0.271	0.037	[−0.354, −0.182]	54.64
Indirect effect	Sports participation → emotional intelligence → self-esteem → school bullying	−0.016	0.005	[−0.021, −0.012]	3.23
	Sports participation → emotional intelligence → school bullying	−0.144	0.024	[−0.201, −0.087]	29.03
	Sports participation → self-esteem → school bullying	−0.065	0.008	[−0.056, −0.092]	13.10
Total mediating effect		−0.225	0.033	[−0.311, −0.141]	45.36

SE, standard error; CI, confidence interval.

## Discussion

### From the perspective of the relationship of personal background with sports participation, school bullying, emotional intelligence and self-esteem in college students

It was found in this study that male college students pay more attention to sports participation than female college students. Male students like to prove their masculinity, so they pay more attention to increasing muscle mass. Male students also tend to have better physical fitness and sport skills than female students, they have relatively better self-disciplined weight control efficiency than female students, and thus rank higher than female students in sports participation (Ouyang et al., 2021).

It was also found in this study that urban college students have better emotional intelligence and self-esteem than rural college students, and rural college students are more likely to be bullied in school than urban college students. This result is consistent with previous studies. Compared with urban college students, rural college students generally have lower emotional intelligence and self-esteem, and are weaker in interpersonal communication, thereby making them more likely to suffer from school bullying (Huang et al., 2017; Lu et al., 2017; Peachey et al., 2017; Canas et al., 2020b; Zhao and Zhou, 2021). From this we can see that emotional intelligence and self-esteem have a certain influence on school bullying.

In addition, significant differences were found between BMI and sports participation and school bullying in this study. The normal weight group’s participation in sports was higher than the other two groups, because normal weight subjects need to spend more time participating in sports so as to achieve or maintain the “ideal body shape” (Ouyang et al., 2021). Moreover, normal weight subjects suffered less school bullying than the other two groups, because underweight and overweight subjects were more likely to suffer from bullying due to their body shape and physical fitness (Shang et al., 2020). Furthermore, underweight and overweight subjects had less sports participation than normal weight subjects. According to the Embodied Cognition Theory, participation in sports can change individuals’ emotional feelings, reduce rebellious teenage behavior, and enhance their self-control ability, which is an important factor in improving peer relationships and further reducing the occurrence of school bullying (Zhu et al., 2016; Xie C. Y. et al., 2019; Wang, 2021). Therefore, sports participation has certain influence on school bullying.

### From the perspective of structured path model of sports participation, school bullying, emotional intelligence and self-esteem

#### Influence of sports participation on school bullying

This research found that sports participation has a significant negative effect on school bullying of college students. This result shows that the higher the degree of sports participation among college students, the less school bullying will occur, and vice versa. This is generally consistent with the research of scholars Demissie et al. (2014), Ren et al. (2014); Moore et al. (2019), and Wang (2021). Some studies (Deater-Deckard et al., 2012; Pietromonaco and Collins, 2017; Yin et al., 2018; Pietromonaco and Beck, 2019; Wang, 2021) hold that sports participation can improve the executive function of bullies and their attention to hostile information and individual risk behaviors (such as smoking and bullying). The quality of their peer relationships will also improve accordingly, thus reducing the occurrence of school bullying. At the same time, sports participation can also improve the cognitive flexibility and emotional adjustment ability of the bullied, enabling the bullied to better emerge from the negative impact of bullying experiences, and finally eliminate the negative impact of school bullying.

Some studies also believe that (Zhu et al., 2016, 2017; Moore et al., 2019) sports participation can effectively improve the emotion regulation ability and belief of both the bullies and the bullied, and promote individuals’ emotion regulation strategy and mental health, thus reducing the deviant behavior of teenagers and enhancing their individual self-control ability. As one aspect of executive function, it is also an important factor to improve peer relationships and further reduce the occurrence of school bullying. In addition, some studies also believe that (Yin et al., 2014; Holbrook et al., 2020; Wang, 2021) regular participation in group sports (such as football, basketball and rope skipping) can help reduce school bullying. In group sports, individuals need to cooperate and interact with others and abide by the specific rules of sports. At the same time, they also need to restrain unreasonable behaviors and assume certain roles and responsibilities to achieve team goals. This serves to greatly improve an individual’s executive function, peer interaction and emotions. Therefore, sports participation is an important factor to reduce school bullying among college students, and there is a reverse correlation between them.

#### Mediating effect of emotional intelligence

This research found that sports participation has a significant positive effect on emotional intelligence. The study results show that people with higher sports participation tend to have higher emotional intelligence, while those with lower sports participation tend to have lower emotional intelligence. This is overall consistent with the findings of Gáspár et al. (2017); Luis Ubago-Jimenez et al. (2019), and Mendez-Gimenez et al. (2022). Emotional intelligence level is related to the degree of sports participation, and participation in any type of sports can improve the level of emotional intelligence and emotional management ability of individuals. This result also supports the research findings of Qian and Zhang (2017); Costa and Fernandes (2019), and Lott and Turner (2019), which shows that in comparison with college students with lower sports participation, those with higher sports participation tend to have higher emotional intelligence and emotional management capabilities. This is due to the emphasis that is placed on aspects of interpersonal relationships such as respect, sharing, cooperation, and concern in sports.

These values further cultivate students’ interpersonal skills, and enhance their emotional intelligence level and emotional management ability. Meanwhile, group sports participation is still better for improving emotional intelligence. This research also found that emotional intelligence has a significant negative effect on school bullying, which shows that bullying among college students will decrease along with an increase in individuals’ emotional intelligence level. The results of this research provides further evidence to the studies of Peachey et al. (2017), Trigueros et al. (2020), and Zhang S. S. et al. (2021), indicating that emotional intelligence has a reverse predictive effect on school bullying. The higher people’s emotional intelligence, the stronger their personal social skills are, making them more equipped and less vulnerable. Similarly, bullies will be less likely to bully others, thus reducing the occurrence of school bullying. At the same time, some scholars believe that (Canas et al., 2020a,b; Leon-del-Barco et al., 2020; Franzen et al., 2021) emotional intelligence is a protective factor of school bullying, and has a key role to play in promoting students’ emotional adjustment and understanding ability.

The emotional intelligence of both the bullies and the bullied is lower than those who are not bullied. In addition, this research also found that sports participation not only can directly negatively affect school bullying, but can also indirectly negatively affect school bullying through the mediating role of emotional intelligence. Some studies (Costa and Fernandes, 2019; Benitez-Sillero et al., 2021; Wang, 2021; Zhang S. S. et al., 2021) have pointed out that sports participation not only can adversely affect the occurrence of school bullying, but can also negatively affect the occurrence of school bullying among college students through emotional intelligence. To sum up, with regard to the Bootstrap mediating effect test procedure, we can infer that emotional intelligence plays a mediating role between sports participation and school bullying.

#### Mediating effect of self-esteem

This research found that sports participation has a significant positive effect on self-esteem. The result shows that the higher the degree of sports participation, the higher the level of self-esteem, and vice versa. This result is consistent with previous researches (Guddal et al., 2019; Qurban et al., 2019; Yan et al., 2019; Bang et al., 2020; Ouyang et al., 2020). Sports participation has a positive correlation with self-esteem. People with higher sports participation will have higher self-esteem, while those with lower sports participation will have lower self-esteem. This research also found that self-esteem has a significant negative impact on school bullying. The result shows that with the improvement of an individual’s self-esteem, he is less prone to suffer from school bullying; on the contrary, along with a decrease in individual self-esteem, one is more likely to become a victim of school bullying. Some studies have pointed out that (Malecki et al., 2015; Tang et al., 2018; Yan et al., 2019; Li and Hu, 2020) self-esteem has a reverse predictive effect on school bullying.

If an individual has low self-esteem, then his self-evaluation and self-worth will be relatively low. When faced with bullying, the individual may not be able to resolutely defend his legitimate rights or even his personal dignity, instead he may choose to compromise blindly, which will eventually lead to continuous school bullying. On the contrary, people with higher self-esteem level have a more mature and steady sense of self-evaluation and self-worth, and their interpersonal skills and their management of negative emotions are better than those with lower self-esteem. It is precisely interpersonal skills and the management of negative emotions that can promote the improvement of peer relations, thus preventing the occurrence of school bullying. Also, some studies have pointed out (Zhao et al., 2018; Wang, 2021) that self-esteem is a preventative factor against school bullying, and the probability of school bullying can be reduced as the self-esteem level of individuals improves.

This study additionally found that sports participation not only can directly and reversely affect school bullying, but also indirectly and reversely affect school bullying through the mediating role of self-esteem. Sports participation can effectively regulate neurotransmitter and hormone levels and improve brain executive function. At the same time, it can promote an individual’s emotion recognition ability, self-esteem and personality development (Ren et al., 2014), thus reducing the occurrence of school bullying. Scholars Yan et al. (2019) and Wang (2021) also agreed that sports participation is a means of preventing school bullying. By increasing sports participation, the level of self-esteem and self-confidence of individuals can be improved, thus improving peer relationship and reducing the occurrence of school bullying. To sum up, according to Bootstrap mediating effect test procedure, the second hypothesis can be inferred valid, which shows that self-esteem plays a mediating role between sports participation and school bullying.

#### Chain mediating effect of emotional intelligence and self-esteem

In this research, the results of the Bootstrap mediating effect test program demonstrate that sports participation has an impact on the occurrence of school bullying among college students through emotional intelligence. Emotional intelligence, on the other hand, has an impact on the school bullying of college students through the mediating role of self-esteem, which verifies the third hypothesis. Emotional intelligence and self-esteem play a chain mediating role between sports participation and school bullying, which means that those with higher sports participation tend to have higher emotional intelligence, and higher emotional intelligence will be accompanied by higher self-esteem. Emotional intelligence and self-esteem are protective factors for individual mental health and help prevent school bullying, thereby reducing the occurrence of such incidents.

On the contrary, those with low sports participation have relatively low emotional intelligence and self-esteem, and are also relatively weaker in regards to interpersonal communication, emotional management and peer relationship, thus increasing the risk of school bullying. The above studies have confirmed that emotional intelligence and self-esteem act as mediators between sports participation and school bullying, and previous studies (Ding and Ma, 2013; Zeng and Hu, 2017; Perez-Fuentes et al., 2019; Garaigordobil, 2020; Li, 2020; Schoeps et al., 2021) have affirmed the positive correlation between emotional intelligence and self-esteem, that is, higher emotional intelligence has a positive impact on individual self-esteem, while lower emotional intelligence has a negative impact on individual self-esteem. Therefore, sports participation adversely affects the occurrence of school bullying among college students through emotional intelligence and self-esteem.

In short, sports participation can not only directly affect the occurrence of school bullying, but also indirectly affect the occurrence of school bullying through the mediating role of emotional intelligence and self-esteem, and the chain mediating role of emotional intelligence and self-esteem. This provides relevant theoretical research basis for reducing school bullying among college students. At the same time, it also provides an effective practical reference to understand how college students’ sports participation, emotional intelligence and self-esteem can reduce school bullying.

## Limitation and conclusion

### Limitation

There are still some limitations in this study. Firstly, this is a cross-sectional study, so the causal relationship between variables cannot be determined. For future studies, a longitudinal tracking strategy can be used to explore the more explicit causal relationship between the variables, which is convenient for further testing the chain mediating model proposed by this study. Secondly, the subjects of this study were university students in Southwest China, but it is hoped that a wider group of subjects can be selected for future research to test the external validity of these results. Finally, this study only considered the general effects of sports participation, emotional intelligence, and self-esteem on school bullying.

However, it did not specifically note participants’ sexual orientation, gender identity and/or gender atypicality, or the influence of homophobia or transphobia on school bullying. Due to sexual identity (real or perceived) being one of the main motivations for bullying in schools, LGBTQIA + individuals or those who are considered gender atypical may be more reluctant to participate in certain types of sports (such as gay men not preferring to playing soccer, etc.). Questions regarding the role these differences play and the effect they may have remain unanswered. It is hoped that future research can consider the sexual orientation and gender identity of the respondents as research variables to further explore the issue of campus bullying and improve the shortcomings of this research.

### Conclusion

Sports participation has a significant positive predictive effect with emotional intelligence and self-esteem, and a significant negative predictive effect with school bullying. Emotional intelligence has a significant positive predictive effect with self-esteem, while having a significant negative predictive effect with school bullying. Moreover, self-esteem also has a significant negative predictive effect with school bullying. The mediating role of emotional intelligence and self-esteem in sports participation and school bullying of college students is confirmed, and this is true for the chain mediating role of “emotional intelligence + self-esteem” in sports participation and school bullying among college students.

Sports participation not only directly affects the occurrence of school bullying, but also indirectly affects the occurrence of school bullying through the mediating role of emotional intelligence and self-esteem and the chain mediating role of emotional intelligence and self-esteem. These results further confirm the Embodied Cognition Theory that exercise is needed for individual emotion regulation, and also prove the predictive effect of emotional intelligence and self-esteem on school bullying. It is hoped that in the future, besides attaching greater significance to college students’ sports participation, it will become necessary for schools to provide courses aimed at developing students’ emotional intelligence, to improve their emotional intelligence and self-esteem, and eventually reduce the occurrence of school bullying.

## Data availability statement

The original contributions presented in this study are included in the article/supplementary material, further inquiries can be directed to the corresponding author.

## Ethics statement

The studies involving human participants were reviewed and approved by the Southwest University. The patients/participants provided their written informed consent to participate in this study.

## Author contributions

OY and LuJ carried out the protocol and questionnaire survey and revised the draft. TJ and PJ recruited the students enrolled in Southwest of China. WK and LiJ undertook the statistical analysis and graphical representation of the database. All authors designed this study, contributed to the article, and approved the final manuscript.
